# Salivary gland ultrasound in the diagnostic workup of juvenile Sjögren’s syndrome and mixed connective tissue disease

**DOI:** 10.1186/s12969-020-00437-6

**Published:** 2020-06-09

**Authors:** Manuela Krumrey-Langkammerer, Johannes-Peter Haas

**Affiliations:** German Center for Pediatric and Adolescent Rheumatology (GCPAR), Gehfeldstr. 24, D-82467 Garmisch-Partenkirchen, Germany

**Keywords:** Juvenile Sjögren’s syndrome, Mixed connective tissue disease, Secondary Sjögren’s syndrome, Ultrasound, Salivary gland ultrasound

## Abstract

**Background:**

Juvenile Sjögren’s Syndrome (jSS) is a rare phenomenon that may appear as primary jSS or associated with mixed connective tissue disease (MCTD) and other autoimmune diseases as secondary jSS. With currently no standard diagnostic procedures available, jSS in MCTD seems to be underdiagnosed. We intended to describe and identify similar distinct salivary gland ultrasound (SGUS) findings in a cohort of primary and secondary jSS patients, focusing on sicca like symptoms and glandular pain/swelling in the patients‘history.

**Methods:**

We present a single-center study with chart data collection. B-mode examinations of salivary glands were obtained with a linear high-frequency transducer and evaluated using the scoring-system of *Hocevar*. Inclusion criteria were: (i) primary or secondary jSS and/or (ii) diagnosis of MCTD and additionally (iii) any presence of sicca like symptoms or glandular pain/swelling.

**Results:**

Twenty five patients with primary (pjSS) and secondary jSS (sjSS) were included in the study (*n* = 25, 21 female, 4 male), with a median age of 15.3 years at the time of first visit and a mean disease duration of 4.9 years. Pathologic SGUS findings were observed in 24 of 25 patients, with inhomogeneous parenchymal appearances with hypoechoic lesions present in 96% of patients. At least one submandibular gland was affected in 88.5% of the whole group, and all patients in the MCTD-group. Twenty of twenty five patients were scanned and scored on a second visit. Pre-malignancies or mucosa-associated lymphoid tissue (MALT) were detected in biopsies of three patients (Hocevar scoring of 40, 33, and 28).

**Conclusion:**

SGUS in patients with pjSS and sjSS is a helpful first-line tool to detect and score salivary gland involvement, in particular when keratoconjunctivitis sicca, xerostomia, or glandular swelling occurs. Juvenile MCTD patients have a significant risk of developing secondary jSS. We propose SGUS as a method in the diagnostic workup and screening for inflammatory changes. Further studies have to determine the predictive value of SGUS for follow up.

## Background

Sjögren’s Syndrome (SS) has originally been described as a chronic autoimmune disease affecting the lacrimal and salivary glands. Primary SS (pSS) is nowadays understood to be a systemic autoimmune disease involving the lungs, kidneys, skin and thyroid gland as well [1]. Moreover, adult and pediatric patients with pSS are known to have a higher risk of developing mucosa-associated lymphoid tissue (MALT) or B-cell lymphomas [2–4].

Xerostomia and Xeropthalmia (keratoconjunctivitis sicca) resulting from glandular involvement observed in other systemic autoimmune diseases such as systemic lupus erythematosus (SLE), mixed connective tissue disease (MCTD), rheumatoid arthritis (RA), dermatomyositis (DM), scleroderma (Sc), sarcoidosis, are termed “secondary” (sSS) or “associated” SS [5–8]. In pediatric patients, both forms of juvenile SS (jSS) have rarely been observed. Reports of jSS in the course of MCTD are limited to a few cases [9]. Studies investigating the pathogenesis of autoimmune focal sialadenitis as present in Sjögren’s syndrome suggest local immune responses to be closely linked to the systemic manifestations of SS with aberrant B-cell activity as a key player [10].

This study aimed to characterize salivary gland ultrasound (SGUS) findings in jSS using a standardized scoring system, thus describing SGUS as a suitable tool for diagnostic workup in patients with any clinical glandular symptoms. Based on our own experiences of SGUS in pjSS [11], we chose the semiquantitative scoring system of Hocevar et al. [12, 13] for its feasibility and accuracy [14]*.*

## Methods

This single-center, cross-sectional cohort study includes SGUS morphologic findings and data collected from patients’ charts admitted between May 2014 and March 2017. As for an observational study based on routine data, only informed written consent was required for participation from both patients/parents and healthy controls.

Included were patients with any clinical glandular symptoms or sicca symptoms who fulfilled the classification criteria for primary (pjSS) or secondary juvenile SS (sjSS) according to the American-European Consensus Group (AECG) [15] and the proposed diagnostic criteria for jSS [16]. Patients with mixed connective tissue disease (MCTD) additionally fulfilled the criteria of Alarcon-Segovia [17, 18].

Data extracted from the patients` charts included demographics, age at onset, clinical symptoms, AECG criteria, antibody constellation, ophthalmologic evaluation of dry eyes, involvement of salivary glands, and their imaging in SGUS, duration of the disease, and histopathologic findings in biopsies if performed. The EULAR Sjögren’s syndrome disease activity index (ESSDAI, [19]) was calculated by using the modified procedure [20] (see Table 1).

**Table 1:** Clinical and laboratory findings

	pjSS (***n*** = 9)	sjSS (***n*** = 6)	sJSS MCTD (***n*** = 10)
“Sicca symptoms”	n = 9 *(100%)*	*n* = 3 (50%)	n = 9 *(90%)*
Glandular swelling	n = 7 (77.8%)	n = 3 (50%)	*n* = 5 (50%)
Glandular pain	n = 4 (44.4%)	n = 2 (33.3%)	n = 6 (60%)
Affected Glandulae submandibulares	n = 8 (88.8%)	n = 5 (83.3%)	n = 10 (100%)
Renal manifestations	*n* = 0	n = 1 (16.6%)	n = 0
Articular	n = 7 (78%)	n = 6 (100%)	n = 10 (100%)
Peripheral nervous system	n = 1 (11.1%)	n = 0	n = 0
Cutaneous	n = 5 *(55.5%)*	n = 5 *(83.3%)*	n = 6 *(60%)*
Fatigue	n = 8 *(88.8%)*	n = 6 *(100%)*	n = 9 *(90%)*
Fever	n = 3 *(33.3%)*	n = 4 *(66.6%)*	n = 6 *(60%)*
ESSDAI (mean)	19.2 (range 7–35)	21.3 (range 10–44)	24.1 (range 3–72)
Remarkable CRP/ ESR (C-reactiv-protein/ erythrocyte sedimentation rate)	n = 8 *(88.8%)*	n = 4 *(66.6%)*	n = 4 *(40%)*
anti-Ro antibody +	n = 9 (100%)	n = 4 (66.6%)	n = 1 (10%)
anti-La antibody +	n = 8 (88.8%)	*n* = 4 (66.6%)	n = 0

SGUS included a B-mode scan during the diagnostic visit, performed with a linear high-frequency transducer (6–15 MHZ, Loqic S8, General Electrics). However, not in all cases was the SGUS performed precisely at times of disease flare (as indicated by divergent ESSDAI scores).

**Fig. 1:**
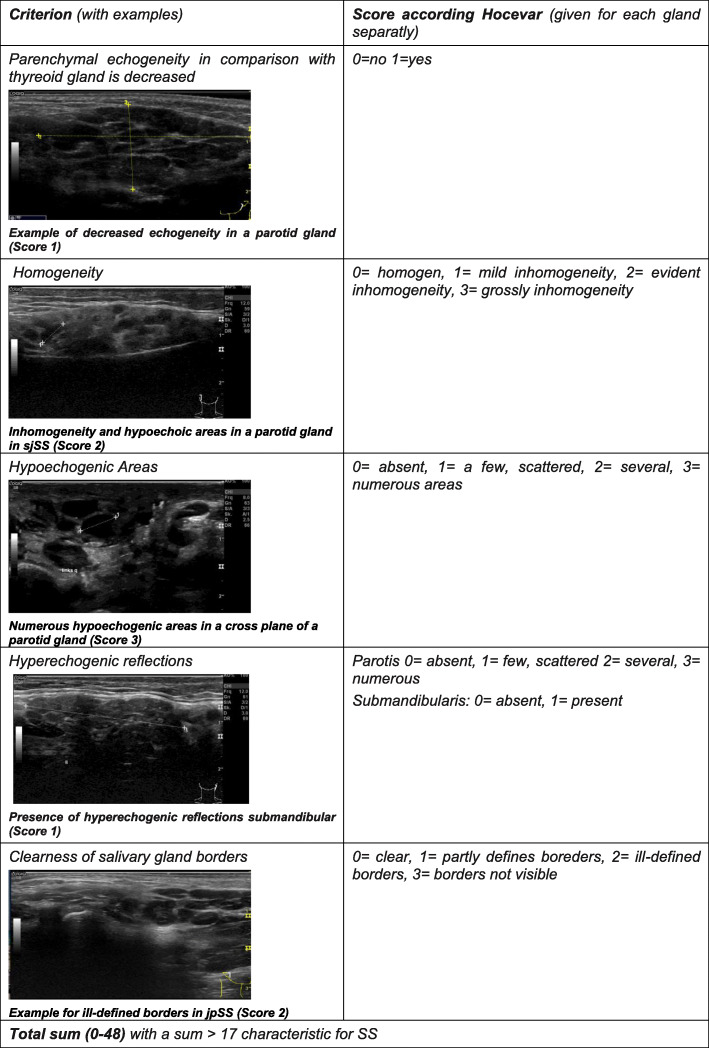
SGUS (salivary gland US) findings- scoring system according A. Hocevar

All ultrasonographic scans were exclusively performed by two experienced examiners in a coadjutant manner (examination performed by one examiner only, followed by discussion and scoring by both examiners) and stored digitally [12–14]. The scans were scored from images, and scores were determined at re-evaluation of the stored image material. The parotid and submandibular glands were scanned bilaterally in a longitudinal and cross plane. All 25 patients with pSS and sSS were graded using the glandular scoring system proposed by Hocevar, a 48-point scale of five visible, non-physiological changes in salivary glands. (See examples in Fig. 1) Within six to 36 months after the first scoring of SGUS, 19/25 patients were scored at a second visit.

B-mode findings were defined as suspicious for pre-malignancy, e.g., if glandular borders were disturbed, parenchymal inhomogeneity was high (score 3) in a nodular area with an irregular border. Consequently, biopsy of these suspicious areas was recommended.

At first, all 25 jSS patients were divided into two groups pjSS (*n* = 9) and sjSS (*n* = 16). Within the sjSS patients, we were able to define a group of ten patients suffering from sjSS associated with MCTD and six patients with other underlying diseases. These three groups (pjSS, sjSS other than MCTD and sjSS with MCTD) were analyzed according to clinical symptoms, SGUS criteria, and Hocevar based score sums. We evaluated and compared score sums in the whole group as well as the subgroups, and in an age-matched healthy control.

Data was extracted into descriptive statistics (mean and range of age, disease duration, clinical and laboratory findings, and distribution of SGUS score). Frequencies were used for categorical variables, Bonferroni correction applied in cases of a significant *p* value (T-test (two-tailed, not paired, equal variance)).

## Results

Of the 41 patients screened,25 fulfilled the inclusion criteria and were enrolled in the study with pjSS (*n* = 9) and sjSS (*n* = 16). The group of sjSS included patients with SLE (*n* = 4) and juvenile idiopathic arthritis (*n* = 2). The MCTD group was singled out because of its unexpected high number and marked similarities. The cohort had an asymmetric distribution of gender (*n* = 21 female, n = 4 male), a mean age of onset of 15.3 years (range 4.2 to 18 yrs), a median age at screening visit of 15.4 yrs. in pjSS and 15.3 yrs. in sjSS and a mean disease duration of 4.9 years (range 0.5 to 15.8 yrs) with mean disease duration of 5.65 yrs. in pjSS, 5.59 yrs. in sjSS without MCTD and 3.43 yrs. in MCTD patients.

All 25 patients were ANA positive (range 1:160 to 1:81290), with titers higher than 1:5120 in 18 cases. SS-A (anti-Ro)-antibodies were found in 9/9 pjSS, and 5/16 sjSS, SS-B-(anti-La) antibodies in 8/9 pjSS, and 4/16 sjSS, IgM-RF (rheumatic factor) in 8/9 pjSS and 9/16 sjSS and immunoglobulin (IgG) levels were increased in 4/9 pjSS and 8/16 sjSS-patients. None of our patients had a decrease in C4 complement. ESSDAI scores were ranging from 3 to 72, with a mean of 19.2 in pjSS, 21.3 in sjSS, and 24.1 in MCTD.

All four male patients belonged to the subgroup of sjSS and U1-RNP-antibody positive MCTD.

As shown in Table 1,21 (81%) of the patients presented “sicca” symptoms (keratoconjunctivitis sicca, xerostomia) at their first visit with differences in the three subgroups (9/9 pjSS, 9/10 MCTD and 3/6 in the remaining sjSS. Glandular swelling was present in 77.8% (*n* = 7) of pjSS patients, but in only 50% (*n* = 8) of sjSS patients (incl. MCTD).

While only 78% of pjSS patients reported arthralgia, all sjSS patients had arthralgia and/or arthritis.

Confirmative biopsies were taken from eight patients, five with pjSS and three with MCTD and sjSS.

### Changes found in jSS using SGUS

Typical morphologic changes visible in B-mode scans of SGUS, as described in adult SS patients, could be demonstrated in both groups (pjSS and sjSS) (see Table 2). Inhomogeneity of parenchyma with several hypoechoic areas was detected in 24/25 patients. Hypoechogenic areas of variable size (range from 2 to 9 mm single lesion) with typical round, scattered manner were seen more frequently in pjSS. These lesions showed a tendency to enlarge and to converge in all cases reaching a score value of 3.

**Table 2:** Positivity according the criteria of the Hocevar score in three different groups in diagnostic workup

Criterion	pSS (*n* = 9)	sSS (*n* = 6) other	sSS (*n* = 10) MCTD	All (*n* = 25)	Healthy control, (*n* = 25)
Decrease in echogenity	3 (33.3%)	3 (50%)	2 (20%)	8 (32%)	0
Inhomogenous parenchyma	9 (100%)	6 (100%)	9 (90%)	24 (96%)	7 (28%)
Hypoechoic areas	9 (100%)	6 (100%)	9 (90%)	24 (96%)	4 (16%)
Hyperechoic reflexes	7 (77.7%)	6 (100%)	8 (80%)	21 (84%)	1 (4%)
Disturbed border	4 (44.4%)	2 (33.3%)	1 (10%)	7 (28%)	0
Hocevar sums	26 (mean) 17–40(range)	12.8 (mean) 4–24(range)	19.7 (mean) 6–29(range)	20.3 (mean) 4–40(range)	0.8 (mean) 0–4(range)
Patients score > 17 (%)	9 (100%)	2 (33%)	8 (80%)	19 (76%)	0

Involvement of at least 3 of 4 salivary glands was detected in 8/9 pjSS, 5/6 sjSS, and 7/10 MCTD patients. Morphologic changes in at least one submandibular gland were present in 88.5% of all patients and 100% within the MCTD-group.

Comparing the score sums, pjSS patients (*n* = 9) showed a mean score of 26 (min = 17, max = 40), while sjSS patients had a mean score of 15.35 (min = 4, max = 29) with only 52.9% exceeding a threshold of 17 (defined to be characteristic for SS). In a control group (healthy) score ranged from 0 to 4 (mean 0.8).

Four patients with MCTD showed a significant homogeneous increase in echogenicity, where a decrease would have been expected. Hyperechoic reflexes were found to be associated with secondary jSS in 82.3%. Morphologic changes in MCTD associated sjSS showed higher score sums than those in sjSS associated with other diseases, but the threshold of 17 could be confirmed for pjSS.

Analyzing correlations of SGUS score sums with t-test (two-tailed, not paired, equal variance) showed significant differences in the score sums of pjSS versus sjSS other than MCTD (*p* < 0,0017, p* < 0,0085 Bonferroni correction) but not in pjSS versus the MCTD sjSS group. This strengthens the assumption that SGUS changes are more impressive in patients with sjSS associated with MCTD than in the remaining group of sjSS.

No correlation was found comparing pathomorphological SGUS findings in major salivary glands and disease duration.

### Monitoring changes in jSS sialadenitis with SGUS

Data on clinical courses was available in all patients covering a disease duration of 4.45 years (mean; range 5–178 months). The small number of patients did not allow evaluation of a correlation between SGUS and disease activity and/or different treatments. Intraindividual morphologic changes were detectable throughout the observation period.

**Fig. 2:**
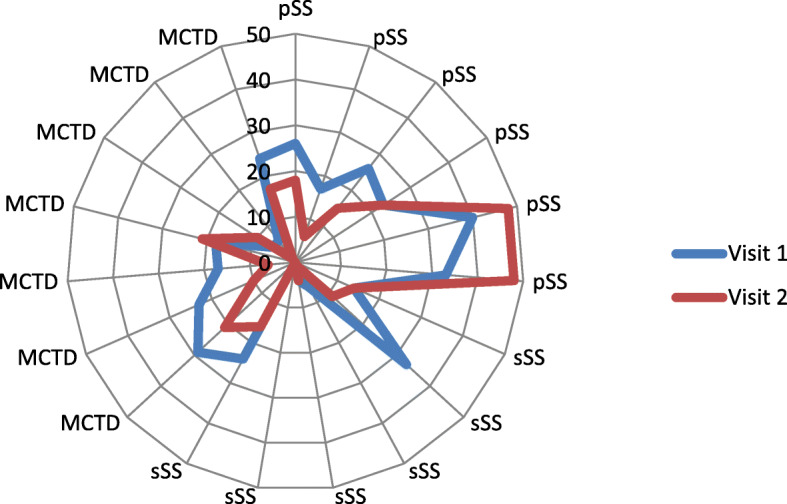
Sum of scoring (acc. Hocevar) in 19 patients at visit 1 and visit 2

Within six to 36 months after the first scoring of SGUS, 19/25 patients were scored at a second visit. As shown in Fig. 2, the SGUS scores dropped in 12 of these 19 patients, while only four patients had higher scores. Four patients showed stable scores. Improvement in scores started in all cases with an improvement of parenchymal homogeneity and a decreased number of scattered hypoechogenic areas, while an increase in scores was due to parotid worsening in all cases (*n* = 2 MCTD, n = 2 pjSS). A multivariant analysis of changes in different domains was not performed due to the small numbers.

### Detecting potential pre-malignancy

In three female patients with pjSS, the macroscopic ultrasonographic changes were suspicious of malignancy, which was confirmed as pre-malignancies (preliminary MALT) from biopsies in all three cases. These biopsies of the parotid glands were performed as a direct consequence of the abnormal US findings (Hocevar scoring of 40, 33, and 28). While two patients (aged 17 yrs. and 18 yrs) developed these changes after a disease duration of 6.8 years and 15.8 years, one patient (aged 12 yrs) had only had symptoms for 6 months.

## Discussion

This study examined ultrasonographic findings in patients with pjSS and sjSS and gland involvement. We highlighted MCTD patients as a subgroup of sjSS. We were able to show SGUS criteria developed and evaluated for adult SS to be applicable in jSS. All patients included in this study demonstrated typical morphologic changes of inhomogeneous parenchymal appearances with hypoechoic lesions. Finally, SGUS was considered to be a useful repeatable tool in detecting morphologic changes suspicious of lymphoproliferative disorders such as MALT.

The scoring system described by Hocevar using five descriptional components for gland echostructure [12, 13] had high accuracy [14] and diagnostic value even compared to scintigraphy [21], although semiquantitative. It is a limitation of our study that only two experienced examinators scored the variables in the 48-point scale of Hocevar in a coadjutant manner. However, the cut-off point of 17 enabled high specificity for jSS in our cohort, particularly in contrast to the healthy controls. We were able to demonstrate its use in the diagnostics and monitoring of jSS as well. Due to the limited number of patients, we did not compare the different scoring systems recently published for adult patients [22].

Abnormalities of the salivary gland’s echostructure seemed to be a hallmark of pSS. The hypoechogenic areas are suggested to represent enlarged glandular lobules that have been replaced by lymphocytic infiltration or fat accumulation [23]. It is of further interest to define whether the hypoechogenicity is due to chronic inflammation with atrophy of these areas. Likely these hypoechoic areas represented areas of non-obstructive sialectasis [24]. In contrast to other studies, we found specific pathological changes in all our pjSS patients when SGUS results were carefully reviewed.

Our study adds some important new aspects to juvenile MCTD, a disorder wherein features of various connective tissue diseases (CTDs) (jSLE, jSc, jDM, jPM) and occasionally jSS can coexist and overlap [7]). Apart from single case studies, there has been no cohort published to date, showing the presence of secondary jSS. This particular cohort was chosen because sjSS in MCTD occurred in our patients more frequently than expected and presented with marked similarities. We found a higher mean score of typical morphologic changes in the MCTD associated sjSS group than we did in the other sjSS patients, although the MCTD group had a shorter disease duration (3.4 vs. 5.59 ys). It has to be emphasized that the changes in this group did not differ in general appearances, but details such as an increase in echogenicity and submandibular gland involvement.

The small sample size did not allow a correlation with disease activity, treatment, and intraindividual SGUS changes. We observed no correlation between pathomorphological SGUS findings in major salivary glands and the duration of symptoms (disease course).

Our data regarding glandular size and vascularization is limited as to our knowledge, no normative data for children and adolescents exists. According to previous data, glands may be enlarged or reduced [23, 25, 26]. Recently Cornec D. et al. [27] showed that Doppler waveform analysis and gland size measurement had poor diagnostic value when compared to salivary gland inhomogeneity.

Although salivary gland biopsy is still the gold standard within the diagnostic criteria, our findings support other authors [28–30] proposing only to perform biopsies when SGUS is negative, and SS has to be confirmed. In this case, SGUS may serve as a supplement, as described earlier.

New data on jSS in comparison to diagnostic criteria in adult SS [1] support our findings of involvement of salivary glands in all our childhood patients with any signs of “sicca” (i.e., keratoconjunctivitis sicca) or involvement of the large salivary glands.

Recently Hammenfors et al. presented a group of 67 jSS patients focusing on SGUS, wherein subjective sicca symptoms were not associated with pathologic SGUS findings [31]. The main difference to our cohort is that all our patients were primarily positive for clinical glandular involvement; additionally, we applied a different scoring system. This is an important consideration when comparing the two groups. It might explain the differences in pathological SGUS findings of 41/67 (but 26/27 out of European patients) compared to 24/25 in our group. There was a difference in primary diagnosis for the sjSS group, as we have seen a remarkable number of MCTD (10/16) patients. In accordance, all patients in the MCTD /SLE subgroup [31] were experiencing major salivary gland swellings, an important fact we would like to highlight. The mean age at inclusion in this multicenter study was higher (16.3y, range 6-25y) than in ours (15.3y, range 4.2 -18y). Hammenfors et al. noted a trend (not significant) to pathologic SGUS findings and experiencing glandular swelling, which we could also confirm [31]. Different from Hammenfors, we were not able to present data of sialometry, sialography, or scintigraphy, but the specific antibodies like anti-Ro/SSA and anti-La/SSB differed somewhat. We found positive anti-Ro/SSA in 56% (vs 88%), and positive anti-La/SSB in 48% vs. 56% with positive SGUS. This might have been due to our larger group of MCTD patients with only one case of anti-Ro/SSA positivity. In conclusion, we agree that findings of pathological SGUS were more common in adolescents than in adults.

A further limitation of our data is that we are were unable to determine the exact time point of the first parenchymal changes in the glands of our patients, visible by utilizing SGUS. All patients included showed specific changes after a mean disease duration of 6.2 years. Overall the data is comparable to the study by Cornec et al. [32], where a mean SD disease duration of the included patients with primary SS was 7.1 to 7.5 years. As jSS is a rare disease, prospective studies addressing this issue will be challenging to conduct.

## Conclusion

In summary, parenchymal inhomogeneity and hypoechoic areas were demonstrated to be the main pathological changes in jSS patients. There was no correlation to the duration of the disease. The study was limited due to its small number of patients.

The five semiquantitative SGUS categories suggested by Hocevar [12, 13] provide an easy to use tool to describe the SGUS morphology and to detect regions suspicious of pre-malignancy, as we have seen in three patients with pjSS after 15.8 years, 6.8 years and 0.5 years duration. These patients showed high aggregate scores in SGUS, especially disturbed gland borders, and afterward preliminary stages of MALT in parotid biopsy. Although the predictive value of SGUS imaging remains to be addressed in prospective studies, it seems a feasible tool in order to screen patients for the presence of salivary gland involvement, especially in patients with juvenile MCTD where secondary jSS is most likely underdiagnosed.

## Data Availability

The data that support the findings of this study are available on request from the corresponding author, [JPH]. The data are not publicly available due to information that could compromise the privacy of research participants.

## Abbreviations

AB: Antibody
AECG: American-European Consensus Group
ANA: Antinuclear antibodies
B-Mode: Brightness mode
C4: Complement factor 4
CFM: Colored flow mapping
CTD: Connective tissue disease
DM: Dermatomyositis
ESSDAI: EULAR Sjögren’s syndrome disease activity index
GCPAR: German Center for Paediatric and Adolescent Rheumatology
IgG: Immunoglobulin G
IgM-RF: Immunoglobulin M rheumatic factor
JIA: Juvenile idiopathic arthritis
jDM: Juvenile dermatomyositis
jPM: Juvenile polymyositis
jMCTD: Juvenile mixed connective tissue disease
jSLE: Juvenile systemic lupus erythematosus
jSc: Juvenile scleroderma
jSS: Juvenile Sjögren’s Syndrome
MALT: Mucosa associated lymphoid tissue
MCTD: Mixed connective tissue disease
MHZ: Mega Hertz
pjSS: Primary juvenile Sjögren’s Syndrome
RA: Rheumatoid arthritis
Sc: Scleroderma
SGUS: Salivary gland ultra-sound
sjSS: Secondary juvenile Sjögren’s Syndrome
SLE: Systemic lupus erythematosus
SS: Sjögren’s Syndrome
U1-RNP: U1-ribonuclein particle
yrs.: Years
